# Simulating A/B testing versus SMART designs for LLM-driven patient engagement to close preventive care gaps

**DOI:** 10.1038/s41746-024-01330-2

**Published:** 2024-11-18

**Authors:** Sanjay Basu, Dean Schillinger, Sadiq Y. Patel, Joseph Rigdon

**Affiliations:** 1 Clinical Product Development, Waymark, San Francisco, CA USA; 2 University of California San Francisco, San Francisco, CA USA; 3 University of Pennsylvania, Philadelphia, PA USA; 4 Wake Forest University, Winston-Salem, NC USA

**Keywords:** Health care, Medical research

## Abstract

Population health initiatives often rely on cold outreach to close gaps in preventive care, such as overdue screenings or immunizations. Tailoring messages to diverse patient populations remains challenging, as traditional A/B testing requires large sample sizes to test only two alternative messages. With increasing availability of large language models (LLMs), programs can utilize tiered testing among both LLM and manual human agents, presenting the dilemma of identifying which patients need different levels of human support to cost-effectively engage large populations. Using microsimulations, we compared both the statistical power and false positive rates of A/B testing and Sequential Multiple Assignment Randomized Trials (SMART) for developing personalized communications across multiple effect sizes and sample sizes. SMART showed better cost-effectiveness and net benefit across all scenarios, but superior power for detecting heterogeneous treatment effects (HTEs) only in later randomization stages, when populations were more homogeneous and subtle differences drove engagement differences.

## Introduction

Health disparities among racial, ethnic, and income groups in the United States are caused, in part, by significant gaps in care, particularly in preventive services^1–3^. To address these gaps, health plans and provider groups are increasingly implementing proactive outreach strategies aimed at bringing patients into care^4,5^. Despite efforts to enhance engagement through A/B testing of phone and text message outreach, engagement rates remain disappointingly low^6–8^. This highlights a critical need for more effective communication strategies that can better cater to the diverse needs of patient populations, particularly in minoritized and underserved communities.

A/B testing, a common approach to optimizing patient engagement among digital health technology and service organizations, involves randomly assigning patients to one of two intervention groups (A or B) and comparing their outcomes; such testing is what a biomedical statistician would refer to as a two-group randomized trial^9^. While useful, this method often requires large sample sizes to detect meaningful differences between groups and is limited in its ability to personalize interventions^10^. Moreover, A/B tests have difficulty providing reliable evidence to decipher heterogeneous treatment effects (HTEs), i.e., identifying whether and why some patients are more or less responsive to different communication strategies^11,12^.

Recent advancements in large language models (LLMs) present a promising avenue to enhance the quantity and quality of personalized outreach. LLMs, driven by their ability to generate persuasive human-like text, are poised to transform patient engagement by enabling more tailored communication at scale^13–15^. However, it is essential to consider the intensity of interventions required to effectively engage patients. Employing human staff is more costly, and the nuances of human interaction are often reserved for patients whose needs are not sufficiently met by automated LLMs^16^. As a result, outreach organizations increasingly employ a tiered approach, with levels of escalating intervention: LLM-generated SMS, LLM-generated voice conversations and, ultimately, human agents^17,18^.

Identifying which patients need each level of engagement has been a challenge for complex, tiered patient outreach pathways. An alternative approach to A/B testing is the Sequential Multiple Assignment Randomized Trial (SMART) design^19,20^. SMART involves randomly assigning patients to different initial interventions, then dynamically re-randomizing subsequent interventions based on the patient’s response. This design allows for the examination of engagement effects along the pathway of intervention adaptation and intensity, rather than merely comparing aggregate outcomes between groups. Previous studies have derived formulae for estimating the minimum sample size necessary for SMART trials to estimate longitudinal and continuous outcome variables in the field of substance use treatment^21^, and using a generalized estimating equations framework to analyze the repeated measures data from such trials^22,23^.

We hypothesize that a SMART design may be useful in deciphering HTEs in patient engagement among digital health technology and service organizations attempting to improve preventive care. We hypothesize that while conventional A/B tests have poor statistical power to detect HTEs in this field despite their widespread use, a SMART design could reliably identify and estimate these effects to guide more personalized and cost-effective outreach strategies. In this study, we develop a microsimulation model to compare the performance of traditional A/B testing versus SMART designs in detecting HTEs and optimizing patient engagement through tiered LLM and human agent outreach. We aim to compare the power between A/B and SMART trial designs at different sample sizes and effect sizes, and estimate the cost-effectiveness for each approach, ultimately informing both the evaluation of potential message strategies, and the value of those strategies when implemented for preventive health outreach at digital health technology and service organizations.

## Results

### Power analysis

**Fig. 1:**
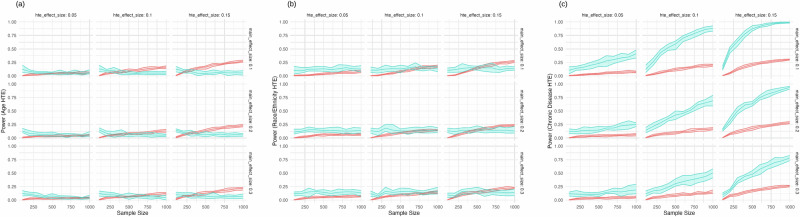
Power vs. sample size. Effect sizes are shown in terms of Cohen’s *d*. Each sample size indicated on the *x*-axis is the sample size of the SMART trial, and the equivalent total of the three A/B trial sample sizes (e.g., a SMART trial of sample size 300 on the *x*-axis corresponds to three A/B trials each of sample size 100): **a** For Age Heterogeneous treatment effect (HTE); **b** For Race/ethnicity Heterogeneous treatment effect (HTE); and **c** For Disease Heterogeneous treatment effect (HTE). Red is A/B testing and blue is SMART trial.

Our power analysis revealed distinct patterns in the ability of A/B testing and SMART designs to detect heterogeneous treatment effects (HTEs) across different sample sizes and effect magnitudes (Fig. 1).

For age-related HTEs (Fig. 1a), both A/B testing and SMART designs demonstrated limited power, rarely exceeding 25% even at the largest sample sizes and effect sizes. A/B testing showed a slight advantage over SMART designs, particularly at larger sample sizes and higher HTE effect sizes. For instance, at a sample size of 1000 and an HTE effect size of 0.15, A/B testing achieved a power of 25.1% (95% CI: 23.7–27.4%) compared to SMART’s 6.7% (95% CI: 2.7–10.0%).

In detecting race/ethnicity HTEs (Fig. 1b), both methods showed modest improvements in power as sample size and effect size increased. SMART designs consistently outperformed A/B testing, albeit marginally. At the maximum sample size of 1000 and HTE effect size of 0.15, SMART achieved a power of 14.7% (95% CI: 9.9–18.0%) versus A/B testing’s 25.7% (95% CI: 23.7–27.0%).

The most pronounced difference between the two methods was observed in their ability to detect chronic disease HTEs (Fig. 1c). SMART designs demonstrated substantially higher power compared to A/B testing, with the difference becoming more apparent as both sample size and effect size increased. At a sample size of 1000 and HTE effect size of 0.15, SMART designs achieved a power of 97.2% (95% CI: 96.0–99.6%), far surpassing A/B testing’s 28.6% (95% CI: 27.1–29.9%).

### False positive rates

Table 1 presents the false positive rates for both methods at a sample size of 1000, across various main effect and HTE effect sizes. For age-related HTEs, both methods maintained false positive rates close to the nominal 5% level, with A/B testing ranging from 8.7% to 12.0% and SMART from 9.6% to 13.3% across different effect sizes.

**Table 1:** False positive rates for A/B testing and SMART designs at a sample size of *n* = 1000

Method	Main effect size (Cohen’s *d*)	HTE size (Cohen’s *d*)	False positive rate for age HTE (95% CI)	False positive rate for race/ethnicity HTE (95% CI)	False positive rate for disease HTE (95% CI)	Net benefit range
A/B	0.1	0.05	0.096 (0.072, 0.130)	0.278 (0.188, 0.320)	0.113 (0.065, 0.158)	−0.038–0.009
SMART	0.1	0.05	0.104 (0.082, 0.151)	0.26 (0.205, 0.351)	0.090 (0.062, 0.126)	0.316–0.522
A/B	0.1	0.1	0.120 (0.075, 0.186)	0.229 (0.177, 0.291)	0.118 (0.052, 0.183)	−0.020–0.039
SMART	0.1	0.10	0.100 (0.062, 0.136)	0.235 (0.158, 0.288)	0.116 (0.079, 0.148)	0.389–0.598
A/B	0.1	0.15	0.102 (0.062, 0.167)	0.271 (0.177, 0.357)	0.115 (0.065, 0.193)	0.026–0.087
SMART	0.1	0.15	0.096 (0.07, 0.128)	0.255 (0.187, 0.29)	0.099 (0.039, 0.182)	0.599–0.654
A/B	0.2	0.05	0.117 (0.067, 0.156)	0.249 (0.190, 0.328)	0.105 (0.055, 0.166)	0.075–0.151
SMART	0.2	0.05	0.104 (0.07, 0.138)	0.235 (0.177, 0.278)	0.117 (0.100, 0.156)	0.505–0.568
A/B	0.2	0.1	0.109 (0.05, 0.189)	0.254 (0.21, 0.318)	0.110 (0.072, 0.158)	0.113–0.151
SMART	0.2	0.1	0.106 (0.07, 0.153)	0.273 (0.195, 0.361)	0.119 (0.082, 0.148)	0.537–0.754
A/B	0.2	0.15	0.087 (0.062, 0.118)	0.259 (0.182, 0.333)	0.097 (0.062, 0.146)	0.156–0.206
SMART	0.2	0.15	0.129 (0.09, 0.193)	0.243 (0.175, 0.288)	0.120 (0.082, 0.158)	0.599–0.654
A/B	0.3	0.05	0.095 (0.052, 0.128)	0.257 (0.197, 0.318)	0.107 (0.065, 0.156)	0.216–0.271
SMART	0.3	0.05	0.127 (0.092, 0.16)	0.243 (0.175, 0.308)	0.117 (0.080, 0.160)	0.668–0.724
A/B	0.3	0.1	0.112 (0.062, 0.171)	0.260 (0.161, 0.359)	0.111 (0.075, 0.138)	0.242–0.280
SMART	0.3	0.1	0.107 (0.062, 0.138)	0.278 (0.185, 0.387)	0.123 (0.082, 0.181)	0.703–0.769
A/B	0.3	0.15	0.12 (0.049, 0.178)	0.252 (0.165, 0.326)	0.123 (0.080, 0.150)	0.273–0.327
SMART	0.3	0.15	0.133 (0.112, 0.158)	0.253 (0.197, 0.28)	0.109 (0.070, 0.190)	0.746–0.787

*SMART* sequential multiple assignment randomized trial.

For race/ethnicity HTEs, both methods showed elevated false positive rates, with A/B testing ranging from 22.9% to 27.1% and SMART from 23.5% to 27.8%. The highest false positive rates were observed for the smallest main effect size (0.1) and largest HTE effect size (0.15) combination.

False positive rates for chronic disease HTEs were generally lower than those for race/ethnicity, but still above the nominal level. A/B testing ranged from 9.7% to 12.3%, while SMART ranged from 9.0% to 12.3%.

### Cost-effectiveness analysis

**Fig. 2:**
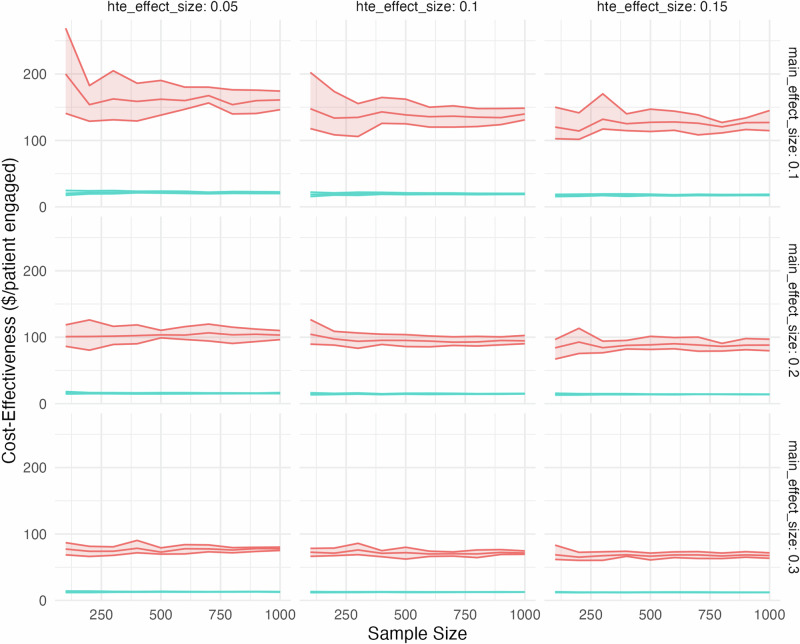
Cost-effectiveness vs. sample size for A/B testing and SMART designs. Each sample size indicated on the *x*-axis is the sample size of the SMART trial, and the equivalent total of the three A/B trial sample sizes (e.g., a SMART trial of sample size 300 on the *x*-axis corresponds to three A/B trials each of sample size 100). Cost-effectiveness on the *y*-axis is in terms of $US 2024 per additional patient engaged. Red is A/B testing and blue is SMART trial.

Our cost-effectiveness analysis (Fig. 2) consistently demonstrated that SMART designs were more cost-effective than A/B testing across all sample sizes and effect size combinations. The incremental cost per additional patient engaged was consistently lower for SMART designs compared to A/B testing.

At a sample size of 1000 and main effect size of 0.1, SMART designs showed a cost-effectiveness of $17.90 (95% CI: $17.32–$18.35) additional spending per additional patient engaged for an HTE effect size of 0.15, compared to $128.10 (95% CI: $126.19–$131.77) for A/B testing. This pattern held across all effect size combinations, with SMART designs maintaining a substantial cost advantage over A/B testing.

The cost-effectiveness gap between the two methods narrowed as the main effect size increased. For instance, at a main effect size of 0.3 and HTE effect size of 0.15, SMART designs achieved a cost-effectiveness of $12.36 (95% CI: $12.14–$12.57) additional spending per additional patient engaged, compared to $67.55 (95% CI: $63.49–$70.99) for A/B testing, representing an 82% cost advantage for SMART designs.

### Net benefit analysis

We conducted a net benefit analysis to further evaluate the relative performance of A/B testing and SMART designs. Across all effect sizes, SMART designs consistently demonstrated higher net benefit compared to A/B testing. At a sample size of 1000 and main effect size of 0.3, the net benefit for SMART designs ranged from 0.668 to 0.787, while for A/B testing, it ranged from 0.216 to 0.327.

The advantage of SMART designs in terms of net benefit became more pronounced as the main effect size and HTE effect size increased. For instance, at a main effect size of 0.3 and HTE effect size of 0.15, SMART designs achieved a net benefit range of 0.746 to 0.787, compared to 0.273 to 0.327 for A/B testing. Even at smaller effect sizes, SMART designs maintained their advantage, with a net benefit range of 0.316 to 0.522 at a main effect size of 0.1 and HTE size of 0.05, compared to −0.038 to 0.009 for A/B testing under the same conditions.

### Counter-intuitive findings and sensitivity analyses

The SMART design’s much higher power in detecting the disease HTE, despite smaller sample sizes in later stages, appeared counterintuitive. Hence, we conducted sensitivity analyses to test two hypotheses (not mutually-exclusive):

1. Targeted population: the SMART design’s adaptive nature allowed for the identification and targeting of subgroups most likely to benefit from specific interventions. In our simulation, the third-stage intervention (LLM vs. human agent) was directed at non-responders from earlier stages, a group potentially enriched for individuals with the disease HTE. This targeted approach enhanced the ability to detect the effect even with a smaller sample size.
2. Reduced heterogeneity: as the SMART trial progressed, responders to earlier interventions were removed, leading to a more homogeneous sample in later stages. This reduction in heterogeneity could increase the power to detect HTEs, even with smaller sample sizes.

To test whether the targeted population (hypothesis 1) or reduced heterogeneity (hypothesis 2) factors were driving the performance differences between the A/B test and SMART trial, we first varied the disease HTE effect size while holding the age and race/ethnicity HTE effect sizes constant at 0.05. As shown in Supplementary Information Fig. 1, the SMART design’s advantage in detecting the disease HTE became dramatically more pronounced as the effect size increased, strongly supporting the “targeted population” factor (hypothesis 1) as a key contributor to the SMART design’s efficiency. At a disease HTE effect size of 0.05, the SMART design already showed a substantial advantage with a power of 72.7% (95% CI: 64.4–79.0%) compared to the A/B test’s 20.1% (95% CI: 18.3–22.3%). This advantage increased markedly as the disease HTE effect size increased. At an effect size of 0.10, the SMART design achieved near-perfect power at 99.9% (95% CI: 99.2–100%), while the A/B test’s power increased only modestly to 33.2% (95% CI: 33.0–33.3%). At the largest effect size of 0.15, the SMART design maintained 100% power, with the A/B test showing no further improvement.

In a second sensitivity analysis (Supplementary Information Fig. 2), the SMART design’s advantage in detecting the disease HTE did not increase as we reduced the heterogeneity of the population characteristics (e.g., age, race, and chronic disease prevalence) in later stages of the SMART trial to mimic the removal of responders. Hence, the SMART trial’s advantage in detecting the disease HTE did not increase as the population became more homogeneous in later stages, rejecting hypothesis 2. These results suggest that the SMART design’s superior performance in detecting disease HTEs is not primarily driven by increased population homogeneity in later stages. Instead, it appears to be more closely related to the design’s ability to target interventions effectively to subgroups most likely to benefit, as demonstrated in our first sensitivity analysis.

## Discussion

This study compared the performance of Sequential Multiple Assignment Randomized Trial (SMART) designs to traditional A/B testing in detecting heterogeneous treatment effects (HTEs) and optimizing patient engagement strategies. Our findings reveal distinct advantages and limitations for each method, depending on the timing of HTEs within the sequential design and the complexity of the effects.

SMART designs demonstrated superior power in detecting HTEs associated with later stages of randomization, particularly as effect sizes increased. This advantage was most pronounced at larger sample sizes, where SMART designs achieved near-perfect power for detecting large HTEs in later stages, while A/B testing showed only modest improvements. The SMART design’s ability to target interventions to non-responders from earlier stages likely contributed to this enhanced performance.

Conversely, A/B testing showed higher power for detecting HTEs associated with earlier stages of randomization, especially at larger sample sizes and effect sizes. This finding suggests that A/B testing’s straightforward approach may be more effective for HTEs that are consistently detectable across the population and do not require complex, multi-stage models to identify.

For HTEs of intermediate complexity or timing, both methods showed similar performance, with SMART designs holding a slight edge. This result indicates that neither method has a clear advantage for detecting HTEs that have moderate interactions with treatment responses across stages. Indeed, the overall levels of power for detecting HTEs were still low, when using parameters in our models that were based on real-world experiments. This result highlights the continued challenge of detecting and personalizing engagement interventions in the healthcare field.

The cost-effectiveness analysis consistently favored SMART designs across all scenarios. This finding suggests that SMART designs may offer a more efficient approach to patient engagement, particularly when substantial HTEs are anticipated in later stages of intervention. The cost-effectiveness of SMART designs could allow for more extensive and frequent patient engagement efforts within the same budget constraints.

However, both methods exhibited elevated false positive rates, especially for HTEs of intermediate complexity. This observation underscores the importance of cautious interpretation of results and the need for robust multiple testing corrections in practice.

Our net benefit analyses indicated that SMART designs provide greater value in terms of correctly identifying responsive patients while minimizing unnecessary interventions. Furthermore, the SMART designs offered a more efficient approach to patient engagement across various scenarios, particularly when heterogeneous treatment effects are anticipated to be substantial. The consistently higher net benefit of SMART designs indicates their potential to improve the overall effectiveness and efficiency of patient engagement strategies.

Our sensitivity analyses provided further insights into the mechanisms driving SMART’s performance. The results supported the hypothesis that SMART’s advantage stems from its ability to target interventions to subgroups most likely to benefit in later stages, rather than from increased population homogeneity as the trial progresses—that is, for detecting heterogeneous treatment effects (HTEs) when populations are more homogeneous and subtle differences drove engagement differences. In our simulation, the last stage of randomization in the SMART was enriched disproportionately with individuals with the disease HTE, which is why SMART had particularly higher power for disease HTE effect size detection versus age or race/ethnicity HTE detection.

These findings have important implications for researchers and practitioners in digital health. When HTEs associated with later stages of intervention are of primary interest, or when adaptive targeting of interventions is feasible, SMART designs may offer substantial advantages. However, for studies focusing on simpler, early-stage HTEs or when adaptive designs are impractical, traditional A/B testing may be more appropriate.

Regarding the anticipated role of LLMs in patient engagement, we envision them as assistive tools for community health workers rather than replacements. The LLM’s primary function would be to augment and enhance the capabilities of human outreach workers, serving as a “co-pilot” in engagement efforts. In practice, this could manifest in several ways: LLMs could draft initial outreach messages for review and personalization by human workers, provide real-time suggestions during conversations with patients, or handle routine inquiries, freeing up human workers to focus on more complex cases or personal interactions. For example, an LLM could generate personalized educational content or appointment reminders, while a human worker could follow up with patients who have specific concerns or require more nuanced support. This hybrid approach leverages the scalability and consistency of LLMs while maintaining the empathy, cultural competence, and complex decision-making abilities of human workers. Importantly, the LLM would be continuously refined based on feedback from both patients and health workers to ensure its outputs remain relevant, accurate, and culturally appropriate.

Limitations of this study include the use of simulated data, which may not fully capture the complexities of real-world patient populations and engagement patterns. Additionally, our implementation of SMART designs and A/B testing represents only one possible approach; alternative implementations may yield different results. Future research should explore the performance of these methods in real-world settings, investigate the impact of different SMART design implementations, and develop strategies to mitigate the elevated false positive rates observed in both methods. Studies examining the long-term outcomes and cost-effectiveness of interventions identified through these methods would also be valuable. A limitation of this study was the use of a brief single-stage LLM-tailored message in each stage of experiment before escalation to the next stage, rather than more complex conversational exchanges which could have provided richer insights into patient concerns and barriers to care. However, due to current US laws (the Telephone Consumer Protection Act 47 USC § 22736), most SMS messages related to healthcare are currently limited to single messages of less than 160 characters unless the patient responds.

In conclusion, this study demonstrates that the choice between SMART designs and A/B testing for detecting HTEs and optimizing patient engagement should be guided by the complexity and timing of anticipated HTEs within the intervention sequence. While SMART designs offer advantages for detecting complex, later-stage HTEs and in overall cost-effectiveness, traditional A/B testing remains valuable for detecting simpler, early-stage HTEs. Researchers should carefully consider these factors when designing studies to optimize patient engagement strategies in digital health interventions.

## Methods

### Overview

This study employs a microsimulation model to compare the performance of traditional A/B testing versus Sequential Multiple Assignment Randomized Trial (SMART) designs in detecting heterogeneous treatment effects (HTEs) and optimizing patient engagement strategies. Our approach consists of five main components:

1. Microsimulation model design: we developed a model that simulates individual patients with various characteristics and their engagement responses to different outreach strategies.
2. A/B test simulation: we simulated a series of traditional A/B tests, each comparing two outreach strategies at a time.
3. SMART design simulation: we simulated a three-stage SMART design that adapts interventions based on patient responses.
4. Power and false positive rate calculations: we assessed the ability of both methods to detect true HTEs and their propensity for false positive results.
5. Cost-effectiveness and net benefit analyses: we evaluated the economic efficiency of both approaches and their overall value in patient engagement.

The microsimulation model incorporates three theorized HTEs: patient age, race/ethnicity, and chronic disease status. These variables were selected based on prior evidence suggesting their potential influence on patient engagement. We simulated populations with varying sample sizes and effect magnitudes to comprehensively compare the performance of A/B testing and SMART designs.

In the following sections, we detail each component of our methodology, including the data sources and parameters used in our simulations, the specific designs of our A/B tests and SMART trials, our analytical approaches, and the metrics used to evaluate performance.

### Microsimulation model design

To examine the statistical power, false positive rates, and cost-effectiveness of traditional A/B testing versus SMART designs in detecting heterogeneous treatment effects (HTEs), we developed a microsimulation model. A microsimulation simulates individual patients, their characteristics (e.g., demographics, clinical history), and their subsequent engagement levels (e.g., responding to outreach) based on the messaging approach used^24^.

We simulated a population with three theorized HTEs: (1) patient age, (2) patient race/ethnicity, and (3) patient chronic disease status. These variables were selected based on prior theory and empirical evidence suggesting their potential influence on patient engagement. The model incorporates these HTEs by assigning different effect sizes to each patient subgroup. The data generating process for all simulations was described using a logistic regression model. The probability of engagement (Pr[engage]) is modeled as a function of the treatment assignment (trt), patient age, race/ethnicity, chronic disease status, and interactions between the treatment and patient characteristics. The process is specified per Eq. (1):

1 $$\begin{array}{l}{{logit}}({Pr }[{{engage}}])={\beta }_{0}+{\beta }_{1}({{trt}}={{empathetic}})+{\beta }_{2}({{trt}}={{human}})\\\qquad\qquad\qquad\qquad+{\beta }_{3}({{trt}}={{weekend}})+ {\beta }_{4}{{age}}+{\beta }_{5}{{race}}+{\beta }_{6}{{chronic}}_{-}{{disease}}\\\qquad\qquad\qquad\qquad+{\beta }_{7}({{trt}}={{empathetic}})\times {{age}}+{\beta }_{8}({{trt}}={{weekend}})\times{{race}}\\\qquad\qquad\qquad\qquad+{\beta }_{9}({{trt}}={{human}})\times {{chronic}}_{-}{{disease}}+\varepsilon \end{array}$$

where Pr[engage] is the probability of engagement; *β*₀ is the intercept (base probability of engagement); *β*_1_, *β*_2_, and *β*_3_ are the main effects of the treatment assignments (empathetic, human, and weekend, respectively); *β*_4_, *β*_5_, and *β*_6_ are the main effects of age, race, and chronic disease status, respectively; *β*_7_, *β*_8_, and *β*_9_ are the interaction/HTE effects between the treatment assignments and patient characteristics; *ε* is the random noise term, which we used to induce correlation within repeated measures by person.

The variables in the model are defined as follows:

- trt: treatment assignment, which can be “empathetic”, “factual”, “human”, “LLM”, “weekday”, or “weekend”.
- age: patient age, a continuous variable in years.
- race: patient race/ethnicity, a categorical variable with levels “White”, “Black”, “Hispanic”, and “Other”.
- chronic_disease: patient chronic disease status, a binary variable (0 = no chronic disease, 1 = any chronic disease).

The interaction terms in the model capture the heterogeneous treatment effects (HTEs) between the treatment assignments and patient characteristics. Specifically:

- *β*_7_ represents the HTE between the empathetic treatment and age, where the effect is present for patients older than 50.
- *β*_8_ represents the HTE between the weekend treatment and race, where the effect is present for patients who are not Black or Hispanic.
- *β*_9_ represents the HTE between the human treatment and chronic disease status, where the effect is present for patients with a chronic disease.

The random noise term (*ε*) is added to the linear predictor to introduce stochasticity in the engagement probabilities.

The engagement probabilities are calculated by applying the inverse logit function to the linear predictor, ensuring that the probabilities are bounded between 0 and 1. Finally, the engagement outcomes are generated by drawing from a Bernoulli distribution with the calculated engagement probabilities based on typical response rates in prior trials, and with the main effect sizes and HTE effect sizes varied across ranges specified further below.

The microsimulation model was calibrated using aggregate patient data and estimates from previous outreach campaigns to ensure realistic effect sizes and variations. The input parameters for the model, including the probability distributions for patient characteristics and engagement risk, are detailed in the Supplementary Information.

### Traditional A/B test simulation

We simulated a series of A/B tests to compare the performance of two outreach strategies at a time: (1) empathetic vs. factual message sentiment, (2) weekday vs. weekend message timing, and (3) LLM-generated vs. human agent outreach. In each A/B test, patients were randomly assigned to one of the two intervention groups, and their engagement outcomes were recorded. Hence, there are three separate A/B trials, each person being assigned to only one intervention, and with only one outcome (whether or not they engage). Each A/B trial has one-third the sample size of the SMART trial described below (e.g., three A/B tests of *N* = 100 each, versus a SMART trial with *N* = 300 to test all three interventions at once, as detailed below).

To test for HTEs, we conducted subgroup analyses by fitting logistic regression models with interaction terms between the intervention group and each theorized HTE covariate (age, race/ethnicity, chronic disease status).

To test for both true positive and false positive HTEs, we fitted the data to the following generalized linear regression model per Eq. (2):

2 $$\begin{array}{l}{{logit}}({Pr }[{{engage}}])={\beta }_{0}+{\beta }_{1}\times I({{trt}}={{intervention}})+{\beta }_{2}\times {{age}}+{\beta }_{3}\times I\times ({{race}}={{Black}})\\\qquad\qquad\qquad\qquad+{\beta }_{4}\times I({{race}}={{Hispanic}})+{\beta }_{5}\times I({{race}}={{Other}})+{\beta }_{6}\times {{chronic}}_{-}{{disease}}\\\qquad\qquad\qquad\qquad+{\beta }_{7}\times I ({{trt}}={{intervention}})\times {{age}}+{\beta }_{8}\times I({{trt}}={{intervention}})\times I ({{race}}={{Black}})\\\qquad\qquad\qquad\qquad+{\beta }_{9}\times I({{trt}}={{intervention}}) \times I({{race}}={{Hispanic}})\\\qquad\qquad\qquad\qquad+{\beta }_{10}\times I({{trt}}={{intervention}})\times I({{race}}={{Other}})\\\qquad\qquad\qquad\qquad+{\beta }_{11}\times I ({{trt}}={{intervention}})\times {{chronic}}\_{{disease}}\end{array}$$

where Pr[engage] is the probability of engagement; *I*(⋅) is the indicator function, which takes the value 1 if the condition inside the parentheses is true and 0 otherwise; trt is the treatment variable, with “intervention” representing the intervention arm and “control” (reference level) representing the control arm for each of the three A/B tests; *β*_0_ is the intercept term; *β*_1_ represents the main effect of the intervention; *β*_2_ represents the main effect of age; *β*_3_, *β*_4_, and *β*_5_ represent the main effects of race (Black, Hispanic, and Other, respectively) compared to the reference level (White); *β*_6_ represents the main effect of having a chronic disease; *β*_7_ represents the interaction effect between the intervention and age; *β*_8_, *β*_9_, and *β*_10_ represent the interaction effects between the intervention and race (Black, Hispanic, and Other, respectively); *β*_11_ represents the interaction effect between the intervention and having a chronic disease.

The statistical significance of these interaction terms (two-tailed *p* < 0.05, without Bonferroni correction for multiple testing to reflect typical practices in the digital health community) was used to determine the presence of HTEs. If any interaction term had a *p* value less than 0.05, we recorded it as detecting the presence of an HTE, and further classified it as a true or false positive.

### SMART design simulation

We simulated a three-stage SMART design (known as a “SMART Design II”^21^) with the following randomizations:

1. Patients were randomly assigned to receive either an empathetic (“Your health matters. Getting a mammogram can give you peace of mind and potentially save your life. We’re here to support you every step of the way”) or factual message (“Regular mammograms are recommended for women over 40. They can detect breast cancer early when it’s easier to treat. Have you scheduled yours?”) sentiment from an LLM chatbot via SMS (LLM sentiment stage).
2. Non-responsive patients were randomly assigned to receive a message at a different day and time (LLM timing stage).
3. Patients who remained non-responsive were randomly assigned to receive personalized outreach from either an LLM (“In [your community], access to mammograms has improved. We can help you find a convenient location and time for your screening”) or a human agent (LLM vs. human agent stage).

The engagement outcomes at each stage were recorded, and the cumulative engagement rate was calculated for each patient subgroup. Hence, each participant in the trial had three outcomes (yes/no to engagement to the first intervention test, non-involvement or yes/no to engagement in the second intervention test, and non-involvement or yes/no to engagement in the third intervention test). For each SMART trial simulation, the sample size was equal to the sum of the sample size of the three separate A/B trials.

To account for the repeated measures in the SMART design, we employed a generalized estimating equation model specified as follows per Eq. (3):

3 $$\begin{array}{l}{{logit}}({Pr }[{{engage}}])={\beta }_{0}+{\beta }_{1}\times I({{trt}}1={{intervention}}1)\\\qquad\qquad\qquad\qquad+{\beta }_{2}\times I ({{trt}}2={{intervention}}2)+{\beta }_{3}\times I ({{trt}}3={{intervention}}3)\\\qquad\qquad\qquad\qquad+{\beta }_{4}\times {{age}}+{\beta }_{5}\times I({{race}}={{Black}}) +{\beta }_{6}\times I({{race}}={{Hispanic}})\\\qquad\qquad\qquad\qquad+{\beta }_{7}\times I({{race}}={{Other}})+{\beta }_{8}\times {{chronic}}\_{{disease}}\\\qquad\qquad\qquad\qquad+{\beta }_{9}\times I ({{trt}}1={{intervention}}1)\times {{age}}+{\beta }_{10}\times I({{trt}}2={{intervention}}2) \times {{age}}\\\qquad\qquad\qquad\qquad+{\beta }_{11}\times I({{trt}}3={{intervention}}3)\times {{age}}+{\beta }_{12}\times I ({{trt}}1={{intervention}}1)\times I({{race}}={{Black}})\\\qquad\qquad\qquad\qquad+{\beta }_{13}\times I ({{trt}}1={{intervention}}1)\times I({{race}}={{Hispanic}})\\\qquad\qquad\qquad\qquad+{\beta }_{14}\times I ({{trt}}1={{intervention}}1)\times I({{race}}={{Other}})\\\qquad\qquad\qquad\qquad+{\beta }_{15}\times I({{trt}}2={{intervention}}2)\times I ({{race}}={{Black}})\\\qquad\qquad\qquad\qquad+{\beta }_{16}\times I({{trt}}2={{intervention}}2)\times I({{race}}={{Hispanic}})+{\beta }_{17}\times I ({{trt}}2={{intervention}}2)\times I({{race}}={{Other}})\\\qquad\qquad\qquad\qquad+{\beta }_{18}\times I({{trt}}3={{intervention}}3)\times I ({{race}}={{Black}})+{\beta }_{19}\times I({{trt}}3={{intervention}}3)\times I({{race}}={{Hispanic}})\\\qquad\qquad\qquad\qquad +{\beta }_{20}\times I({{trt}}3={{intervention}}3)\times I({{race}}={{Other}})+{\beta }_{21}\times I ({{trt}}1={{intervention}}1)\times {{chronic}}\_{{disease}}\\\qquad\qquad\qquad\qquad+{\beta }_{22}\times I ({{trt}}2={{intervention}}2)\times {{chronic}}\_{{disease}}\\\qquad\qquad\qquad\qquad+{\beta }_{23}\times I ({{trt}}3={{intervention}}3)\times {{chronic}}\_{{disease}}\end{array}$$

where Pr[engage] is the probability of engagement; *I*(⋅) is the indicator function, which takes the value 1 if the condition inside the parentheses is true and 0 otherwise; trt1, trt2, and trt3 are the treatment variables for the first, second, and third stages of the SMART design, respectively, with “intervention1”, “intervention2”, and “intervention3” representing the corresponding intervention arms and “control” (reference level) representing the control arm; age, race, and chronic_disease are defined as in the A/B test model; *β*_0_ is the intercept term; *β*_1_, *β*_2_, and *β*_3_ represent the main effects of the interventions at each stage; *β*_4_–*β*_8_ represent the main effects of age, race, and chronic disease, as in the A/B test model; *β*_9_–*β*_11_ represent the interaction effects between the interventions and age at each stage; *β*_12_–*β*_20_ represent the interaction effects between the interventions and race at each stage; *β*_21_–*β*_23_ represent the interaction effects between the interventions and having a chronic disease at each stage.

The GEE model assumes an exchangeable correlation structure to account for the within-subject correlations across the stages of the SMART design^25^.

### Power calculations, false positive rates, cost-effectiveness analysis and net benefit analysis

We simulated A/B tests and SMART designs with sample sizes ranging from 100 to 1000 patients for each of the three A/B trials (i.e., 300 to 3000 for the aggregate SMART trial). For each sample size, we conducted 100 simulations and calculated the proportion of simulations in which a significant HTE was detected (i.e., power) for each of the three HTEs (age, race/ethnicity, chronic disease status).

Additionally, we calculated the false positive rates for each non-existent HTE interaction (the ones not present in the data-generating process) in both A/B tests and SMART designs. For each intervention experiment (or each randomization in the SMART design), we tested if the *p* value of any interaction terms for the non-existent HTEs was less than 0.05 (without correction for multiple testing, given typical practice in the digital health community), indicative of a false positive detection.

We conducted a cost-effectiveness analysis by assigning typical costs to each type of outreach based on industry estimates. The total cost of each simulated trial was calculated, and the incremental cost-effectiveness ratio (ICER) was computed as the additional cost per additional patient engaged compared to the baseline (no intervention) scenario. We assigned typical costs to each type of outreach based on industry estimates, per message (Supplementary Information), including the technology infrastructure, personnel, and other resources required to deliver the interventions. The total cost of each simulated trial was calculated by summing the costs of all outreach attempts based on the number of patients assigned to each intervention.

The effectiveness of each trial design was measured by the proportion of patients engaged compared to the baseline (no intervention) scenario. We calculated the engagement rate for each patient subgroup and the overall population in each simulated trial.

The ICER was computed as the ratio of the difference in costs to the difference in effectiveness between two trial designs^26–28^, representing the additional cost per additional patient engaged when moving from one trial design to another. A lower ICER indicates a more cost-effective approach.

We conducted a Net Benefit Analysis as described by Vickers et al.^29^ to evaluate the clinical utility of our LLM-based intervention. The net benefit was calculated as per Eq. (4):

4 $${{Net}}\,{{Benefit}}=({{True}}\,{{Positives}}/N)-({{False}}\,{{Positives}}/N)\,\ast\, (pt/(1-pt))$$

Where *N* is the total number of patients, *pt* is the threshold probability at which the benefit of intervention is equal to the harm of unnecessary intervention.

We calculated net benefit for a range of main effect sizes (0.1, 0.2, 0.3) and heterogeneous treatment effect sizes (0.05, 0.1, 0.15). For the net benefit analysis, we used threshold probabilities ranging from 5% to 30% to create decision curves.

To assess the impact of varying effect sizes on the power, false positive rates, cost-effectiveness and net benefit of A/B testing and SMART designs, we conducted a sensitivity analysis by simulating trials with different combinations of main effect sizes (Cohen’s *d* values of 0.1, 0.2, and 0.3) and HTE effect sizes (Cohen’s *d* of 0.05, 0.1, and 0.15), representing a range from small to large effects.

### Sensitivity analysis

To assess the robustness of our findings and explore potential mechanisms driving the performance differences between A/B testing and SMART designs, we conducted a series of sensitivity analyses. First, we varied the HTE effect sizes across stages to examine whether the SMART trial’s advantage in detecting the disease HTE becomes more pronounced as the effect size increases in later stages, while keeping the age and race HTE effect sizes constant, providing insight into whether and when the SMART design may be beneficial for detecting HTEs among smaller samples non-responsive to earlier interventions (analysis 1). Second, we modified the population characteristics across stages to assess whether the SMART trial’s advantage in detecting the disease HTE increases as the population becomes more homogeneous in later stages (analysis 2). These sensitivity analyses aimed to provide a more comprehensive understanding of the factors influencing the relative performance of A/B testing and SMART designs in detecting heterogeneous treatment effects, and to assess the robustness of our findings to different assumptions and scenarios.

All simulations and analyses were performed using R (version 4.1.0), and the code is available at https://github.com/sanjaybasu/smart-engagement/. The study was deemed exempt as all patients were simulated and no actual individual patient data were used; informed consent was waived by the Western Institutional Review Board WIRB-Copernicus Group IRB (Reference # 20221442).

## Supplementary information

Supplemental Information

## Data Availability

Data are available without restrictions at https://github.com/sanjaybasu/smart-engagement/.

## Code availability

Code, which can be run in R (version 4.1.0) is available without restrictions at https://github.com/sanjaybasu/smart-engagement/.
