# Supplementary material for: Simulating A/B testing versus SMART designs for LLM-driven patient engagement to close preventive care gaps
Source: NPJ Digit Med. 2024 Nov 18;7:322. doi: 10.1038/s41746-024-01330-2 (PMC11574204; doi:10.1038/s41746-024-01330-2)

**Supporting Information**

The microsimulation model incorporated representative patient data and estimates from previous outreach campaigns to ensure realistic effect sizes and variations.[^27,28^](https://www.zotero.org/google-docs/?vmGTrZ) The model simulates individual patients with characteristics drawn from probability distributions based on real-world data (see detailed code at https://github.com/sanjaybasu/smart-engagement/).

Data Sources and Simulation Parameters

Our microsimulation model was built using data from published literature on health disparities, patient engagement, and clinical trial recruitment. This approach allowed us to create a realistic representation of the patient population while basing our parameters on peer-reviewed research. Below, we provide detailed information on how each patient characteristic was modeled and the data sources used to inform our parameter choices.

Age Distribution

Age was sampled from a uniform distribution between 18 and 90 years. This range was chosen to reflect the typical age range of adults in population health initiatives and clinical trials. The distribution was chosen to allow for adequate representation across all adult age groups, and based on the age distribution reported in Caraballo et al.², which analyzed trends in racial and ethnic disparities in barriers to timely medical care among adults in the US from 1999 to 2018. This comparison ensured that our simulated population was reasonably capturing the broader US adult population seeking medical care.

Chronic Disease Status

Chronic disease status was assigned as a binary value (0 or 1) based on a probability parameter calibrated to the prevalence of chronic diseases in the target population. The probability of having a chronic disease was set to 0.60, based on the following data:

1. Nelson et al.¹, which reported on health equity in preventive services and provided information on chronic disease prevalence.

2. Caraballo et al.³, which analyzed racial and ethnic disparities in access to health care among adults in the United States over a 20-year period.

We chose a slightly higher prevalence than some national estimates to reflect the fact that our simulated population represents individuals engaged with the healthcare system, who are more likely to have chronic conditions than the general population.

Race/Ethnicity

Race/ethnicity was sampled from a categorical distribution with probabilities reflecting the racial/ethnic composition of the target population. The categories and their probabilities were set as follows:

- White: 0.60

- Black: 0.13

- Hispanic: 0.18

- Asian: 0.06

- Other: 0.03

These probabilities were informed by:

1. The racial/ethnic distribution reported in Caraballo et al.² for their analysis of barriers to timely medical care.

2. Lee et al.²⁸, which examined the effects of communication source and racial representation in clinical trial recruitment flyers.

We adjusted the probabilities slightly to ensure adequate representation of minority groups for the purpose of detecting potential race/ethnicity-related heterogeneous treatment effects.

Engagement Risk Calculation

The engagement risk for each patient was calculated using the following logistic regression model:

logit(Pr[engage]) = β₀ + β₁(trt = empathetic) + β₂(trt = human) + β₃(trt = weekend) + β₄age + β₅race + β₆chronic_disease + β₇(trt = empathetic) × age + β₈(trt = weekend) × race + β₉(trt =

Where:

- β₀ is the intercept (base probability of engagement)

- β₁, β₂, and β₃ are the main effects of the treatment assignments

- β₄, β₅, and β₆ are the main effects of age, race, and chronic disease status

- β₇, β₈, and β₉ are the interaction/HTE effects

- ε is a random noise term

The baseline probability (β₀) was set to -3 (on the logit scale), corresponding to a baseline engagement probability of about 5% without any intervention. This was based on typical response rates observed in previous outreach campaigns reported in the literature⁶,⁷,⁸.

Main effect sizes (β₁, β₂, β₃) were varied in our simulations (0.1, 0.2, and 0.3 in terms of Cohen's d), representing small to moderate intervention effects. These effect sizes were chosen based on the range of effects reported in previous studies of patient engagement interventions⁶,⁷,⁸. The lower bound (0.1) was informed by Stewart de Ramirez et al.⁶, who found modest improvements in breast cancer screening rates with engagement interventions. In their study, the odds ratio for mammogram completion in the "Least" intervention group (text messaging only) was 1.83 for Medicaid patients and 1.72 for commercially insured patients, which corresponds to small effect sizes. The middle value (0.2) aligns with findings from Sapre et al.⁷, who observed moderate improvements in engagement with a text messaging platform for youth diabetes prevention. They reported that highly responsive participants (46% of the sample) responded to over 75% of interactive messages sent over 12 weeks, indicating a moderate effect size. The upper bound (0.3) was based on Cherryhomes and Guillot-Wright⁸, who found stronger effects for a text messaging campaign to improve health disparities among migrant workers. They reported odds ratios ranging from 2.33 to 3.08 for different intervention arms, which correspond to moderate to large effect sizes. By using this range of effect sizes, we aimed to capture the variability in intervention effectiveness observed across different contexts and populations in recent patient engagement studies. This approach allows our simulation to explore scenarios from minimally effective interventions to those with more substantial impacts, providing a comprehensive assessment of trial design performance across a realistic spectrum of potential outcomes.

Heterogeneous treatment effect sizes (β₇, β₈, β₉) were also varied (0.05, 0.1, and 0.15 in terms of Cohen's d) to explore a range of potential subgroup differences, informed by the findings of Jennerich et al.²⁷ on heterogeneity of treatment effect in a randomized trial of a communication intervention. In particular, the range encompasses the interaction effect observed by Jennerich et al. between the intervention and patient income (which often correlates with age) on quality of communication ratings. The chronic disease heterogeneous treatment effect sizes are also based on Jennerich et al.'s finding of a significant interaction between the intervention and patient-assessed health status on depression symptoms at 6 months. Their reported interaction effect for patients with poor self-assessed health corresponds to the upper end of our effect size range.

The random noise term (ε) was sampled from a normal distribution with mean 0 and standard deviation 0.01, introducing a small amount of variability to reflect real-world unpredictability in patient engagement.

By using this detailed approach to modeling patient characteristics and engagement risk, we aimed to create a realistic simulation that could effectively test the performance of different trial designs in detecting heterogeneous treatment effects, grounded in the findings of recent, relevant literature.

*Estimation of Resources and Costs*

Our cost estimation for the LLM-based intervention incorporates both fixed and variable costs, reflecting the design of the A/B tests and SMART trial as described in our methods. The cost-effectiveness analysis incorporated realistic cost estimates for each type of outreach based on industry benchmarks and published data.²⁹⁻³³

Fixed Costs:

- Voiceflow platform subscription: $625/month = $7,500/year

- HIPAA compliance markup (20%): $1,500/year

- Total platform cost: $9,000/year

- Personnel costs (annual):

- Project manager (0.25 FTE): $31,250

- User experience designer (0.1 FTE): $11,000

- Quality assurance tester (0.1 FTE): $8,500

- ML/AI engineer or data scientist (0.2 FTE): $30,000

- Total personnel cost: $80,750/year

- Total fixed costs: $89,750/year

Variable Costs:

1. LLM-Generated SMS Outreach:²⁹^,^³⁴

- Empathetic Message Generation: $1.20 per message (based on $0.03 per token for GPT-4o output, 40 tokens)

- Factual Message Generation: $0.60 per message (based on $0.03 per token for GPT-4o output, 20 tokens)

- SMS Sending Cost: $0.03 per message (regardless of weekday or weekend)

2. Human Agent Outreach:³⁰^,^³¹^,^³³

- Phone Call: $2.70 to $5.60 per service encounter (includes agent salaries, benefits, training, and operational expenses)

- SMS: $0.80 per service encounter (assuming 20 SMS interactions per hour, based on $15.96 hourly pay for a customer service agent in 2024)

Cost Calculation for A/B Tests:

Each A/B test compares two strategies, with participants receiving only one message:

1. Empathetic vs. Factual message sentiment:

- Empathetic: $1.20 (LLM) + $0.03 (SMS) = $1.23

- Factual: $0.60 (LLM) + $0.03 (SMS) = $0.63

2. Weekday vs. Weekend message timing: $0.03 (SMS only)

3. LLM-generated vs. Human agent outreach:

- LLM-generated: $1.20 (LLM) + $0.03 (SMS) = $1.23

- Human agent: $5.60 (upper range of phone call cost) + $0.03 (SMS) = $5.63

Cost Calculation for SMART Design:

The SMART design involves up to three stages of intervention, with costs accumulating based on participant responsiveness:

1. LLM sentiment stage: $1.23 per participant (empathetic message cost)

2. LLM timing stage (for non-responders): Additional $0.03 (SMS cost only)

3. LLM vs. human agent stage (for remaining non-responders):

- LLM option: Additional $1.23

- Human agent option: Additional $5.63

All costs are reported in 2024 US dollars. The cost parameters for LLM-generated SMS outreach are based on published pricing information for GPT-4o.²⁹ The cost of sending an SMS message is based on industry averages.³² The cost parameters for human agent outreach are based on industry benchmarks for call center costs and average hourly pay for customer service agents.³⁰^,^³¹^,^³³

It is important to note that the LLM (GPT-4o) simulated in this study was pre-trained and not fine-tuned or subjected to Retrieval-Augmented Generation. While our specific LLM-generated messages were simulated as part of our study design, we based them on examples of empathetic and factual messages typical in health communication.

In our cost-effectiveness analysis, we calculated total costs by combining these fixed and variable components based on the simulated number of participants in each arm of the A/B tests and each stage of the SMART trial, their engagement levels, and the resulting use of additional interventions for non-responders. This approach allows us to capture the cost implications of both trial designs and variable participant engagement patterns across different effect sizes.

The incremental cost-effectiveness ratio (ICER) was calculated as the difference in total costs between the intervention and control groups, divided by the difference in the number of participants engaged. This provides a measure of the additional cost per additional participant engaged due to the intervention.

While our specific LLM-generated messages were simulated as part of our study design, the following examples illustrate the types of empathetic and factual messages that were used as a basis for our LLM to generate similar content:

Empathetic messages:

1. "Your health matters. Getting a mammogram can give you peace of mind and potentially save your life. We're here to support you every step of the way."

2. "We understand that life can get busy, but your health is important. A mammogram takes only a few minutes and can make a big difference. How can we help you schedule one?"

These messages were inspired by the approach taken in Sapre et al.⁷, where they emphasized creating a supportive and motivational tone in their messages to youth.

Factual messages:

1. "Regular mammograms are recommended for women over 40. They can detect breast cancer early when it's easier to treat. Have you scheduled yours?"

2. "Did you know? Mammograms can find breast lumps up to two years before they can be felt. Schedule your screening today."

These messages were modeled after the approach in Stewart de Ramirez et al.⁶, where they used brief, informative content to encourage breast cancer screening.

Personalized messages, as potential second- or third-stage responses or alternatives to escalating to human agents:

1. "As an [age] year old woman, your risk for breast cancer increases. A mammogram is a crucial step in early detection. Can we help you schedule one?"

2. "In [your community], access to mammograms has improved. We can help you find a convenient location and time for your screening."

These messages were designed to address potential HTEs related to age and community access, similar to the approach taken by Cherryhomes and Guillot-Wright⁸ in tailoring messages to their target population.

**References**

[1. Nelson, H. D. *et al.* Achieving Health Equity in Preventive Services: A Systematic Review for a National Institutes of Health Pathways to Prevention Workshop. *Ann. Intern. Med.* **172**, 258 (2020).](https://www.zotero.org/google-docs/?7aLqKE)

[2. Caraballo, C. *et al.* Trends in racial and ethnic disparities in barriers to timely medical care among adults in the US, 1999 to 2018. *JAMA Health Forum* **3**, e223856–e223856 (2022).](https://www.zotero.org/google-docs/?7aLqKE)

[3. Caraballo, C. *et al.* Racial and ethnic disparities in access to health care among adults in the United States: a 20-year National Health Interview Survey analysis, 1999–2018. *MedRxiv* (2020).](https://www.zotero.org/google-docs/?7aLqKE)

[4. Ukert, B., David, G., Smith‐McLallen, A. & Chawla, R. Do payor‐based outreach programs reduce medical cost and utilization? *Health Econ.* **29**, 671–682 (2020).](https://www.zotero.org/google-docs/?7aLqKE)

[5. Rechel, B. How to enhance the integration of primary care and public health? Approaches, facilitating factors and policy options. *Eur. Obs. Health Syst. Policies Policy Brief 10* (2020).](https://www.zotero.org/google-docs/?7aLqKE)

[6. Stewart De Ramirez, S. *et al.* Closing the Gap: A Comparison of Engagement Interventions to Achieve Equitable Breast Cancer Screening in Rural Illinois. *Popul. Health Manag.* **25**, 244–253 (2022).](https://www.zotero.org/google-docs/?7aLqKE)

[7. Sapre, M. *et al.* The Development of a Text Messaging Platform to Enhance a Youth Diabetes Prevention Program: Observational Process Study. *JMIR Form. Res.* **8**, e45561 (2024).](https://www.zotero.org/google-docs/?7aLqKE)

[8. Cherryhomes, E. & Guillot-Wright, S. Dissemination and Implementation of a Text Messaging Campaign to Improve Health Disparities among Im/Migrant Workers. *Int. J. Environ. Res. Public. Health* **20**, 5311 (2023).](https://www.zotero.org/google-docs/?7aLqKE)

[9. Kohavi, R., Tang, D. & Xu, Y. *Trustworthy Online Controlled Experiments: A Practical Guide to a/b Testing*. (Cambridge University Press, 2020).](https://www.zotero.org/google-docs/?7aLqKE)

[10. Miller, A. P. & Hosanagar, K. An empirical meta-analysis of e-commerce a/b testing strategies. *Whart. Sch. Univ. Pa.* (2020).](https://www.zotero.org/google-docs/?7aLqKE)

[11. Larsen, N. *et al.* Statistical Challenges in Online Controlled Experiments: A Review of A/B Testing Methodology. *Am. Stat.* **78**, 135–149 (2024).](https://www.zotero.org/google-docs/?7aLqKE)

[12. Somanchi, S., Abbasi, A., Kelley, K., Dobolyi, D. & Yuan, T. T. Examining User Heterogeneity in Digital Experiments. *ACM Trans. Inf. Syst.* 3578931 (2023) doi:10.1145/3578931.](https://www.zotero.org/google-docs/?7aLqKE)

[13. Selvaskandan, H., Gee, P. O. & Seethapathy, H. Technological Innovations to Improve Patient Engagement in Nephrology. *Adv. Kidney Dis. Health* **31**, 28–36 (2024).](https://www.zotero.org/google-docs/?7aLqKE)

[14. Shah, N. H., Entwistle, D. & Pfeffer, M. A. Creation and adoption of large language models in medicine. *Jama* **330**, 866–869 (2023).](https://www.zotero.org/google-docs/?7aLqKE)

[15. Subramanian, C. R., Yang, D. A. & Khanna, R. Enhancing health care communication with large language models—the role, challenges, and future directions. *JAMA Netw. Open* **7**, e240347–e240347 (2024).](https://www.zotero.org/google-docs/?7aLqKE)

[16. Harris, E. Large language models answer medical questions accurately, but can’t match clinicians’ knowledge. *JAMA* (2023).](https://www.zotero.org/google-docs/?7aLqKE)

[17. Kolasani, S. Optimizing natural language processing, large language models (LLMs) for efficient customer service, and hyper-personalization to enable sustainable growth and revenue. *Trans. Latest Trends Artif. Intell.* **4**, (2023).](https://www.zotero.org/google-docs/?7aLqKE)

[18. Wulf, J. & Meierhofer, J. Exploring the Potential of Large Language Models for Automation in Technical Customer Service. Preprint at http://arxiv.org/abs/2405.09161 (2024).](https://www.zotero.org/google-docs/?7aLqKE)

[19. Collins, L. M., Murphy, S. A. & Strecher, V. The multiphase optimization strategy (MOST) and the sequential multiple assignment randomized trial (SMART): new methods for more potent eHealth interventions. *Am. J. Prev. Med.* **32**, S112–S118 (2007).](https://www.zotero.org/google-docs/?7aLqKE)

[20. Collins, L. M., Nahum-Shani, I. & Almirall, D. Optimization of behavioral dynamic treatment regimens based on the sequential, multiple assignment, randomized trial (SMART). *Clin. Trials* **11**, 426–434 (2014).](https://www.zotero.org/google-docs/?7aLqKE)

[21. Seewald, N. J. *et al.* Sample size considerations for comparing dynamic treatment regimens in a sequential multiple-assignment randomized trial with a continuous longitudinal outcome. *Stat. Methods Med. Res.* **29**, 1891–1912 (2020).](https://www.zotero.org/google-docs/?7aLqKE)

[22. Nahum-Shani, I. *et al.* A SMART data analysis method for constructing adaptive treatment strategies for substance use disorders. *Addict. Abingdon Engl.* **112**, 901–909 (2017).](https://www.zotero.org/google-docs/?7aLqKE)

[23. Nahum-Shani, I., Qian, M. & Almirall, D. Q-Learning: A Data Analysis Method for Constructing Adaptive Interventions - PMC. *Psychol Methods* **17**, 478–94.](https://www.zotero.org/google-docs/?7aLqKE)

[24. Basu, S. Microsimulation. in *Systems Science and Popualtion Health* (Oxford University Press, Oxford, 2015).](https://www.zotero.org/google-docs/?7aLqKE)

[25. d3center - Data Science For Dynamic Intervention Decision-Making Center. d3center-isr/qlaci. *d3center-isr/qlaci* https://github.com/d3center-isr/qlaci (2023).](https://www.zotero.org/google-docs/?7aLqKE)

[26. ISPOR RCT-CEA Task Force Report. Good Research Practices for Cost-Effectiveness Analysis Alongside Clinical Trials: The ISPOR RCT-CEA Task Force Report.](https://www.zotero.org/google-docs/?7aLqKE)

[27. Jennerich, A. L., Downey, L., Engelberg, R. A. & Curtis, J. R. Heterogeneity of treatment effect in a randomized trial of a communication intervention. *J. Pain Symptom Manage.* **64**, 298–303 (2022).](https://www.zotero.org/google-docs/?7aLqKE)

[28. Lee, S., Lee, N. & Kirkpatrick, C. E. Effects of Communication Source and Racial Representation in Clinical Trial Recruitment Flyers. *Health Commun.* **38**, 790–802 (2023).](https://www.zotero.org/google-docs/?7aLqKE)

[29. OpenAI. Pricing. https://openai.com/api/pricing/.](https://www.zotero.org/google-docs/?7aLqKE)

[30. Maestroqa. Call Center Cost Per Call: How to Calculate & Reduce It. https://www.maestroqa.com/blog/call-center-cost-per-call.](https://www.zotero.org/google-docs/?7aLqKE)

[31. LiveAgent. Cost Per Call (Explained). *LiveAgent* https://www.liveagent.com/customer-support-glossary/cost-per-call/.](https://www.zotero.org/google-docs/?7aLqKE)

[32. Virtual Agents in Customer Service: Managing Costs. https://logic2020.com/insight/virtual-agent-customer-service-costs/ (2020).](https://www.zotero.org/google-docs/?7aLqKE)

[33. PayScale. Customer Service Agent Hourly Pay in 2024. https://www.payscale.com/research/US/Job=Customer_Service_Agent/Hourly_Rate.](https://www.zotero.org/google-docs/?7aLqKE)

[34. Lee, Y. K., Suh, J., Zhan, H. & Ong, D. C. Large Language Models Produce Responses Perceived to be Empathic. *arXiv* **2403.18148**, (2024).](https://www.zotero.org/google-docs/?7aLqKE)

35. Vickers A J, Van Calster B, Steyerberg E W. Net benefit approaches to the evaluation of prediction models, molecular markers, and diagnostic tests *BMJ* 2016; 352 :i6 doi:10.1136/bmj.i6

36. 47 U.S. Code § 227 - Restrictions on use of telephone equipment. https://www.law.cornell.edu/uscode/text/47/227 (2011).

**Supplementary Figure 1:** Comparison of the performance of the A/B test and SMART trial under these conditions to see if the SMART trial's advantage in detecting the disease HTE becomes more pronounced as the effect size increases in later stages. We ran simulations with increasing HTE effect sizes for the disease HTE in later stages, while keeping the age and race HTE effect sizes constant. As the SMART trial's advantage in detecting the disease HTE becomes more pronounced as the effect size increases, the results suggest that the targeted population factor contributes to the SMART trial's performance. HTE: Heterogeneous treatment effect.


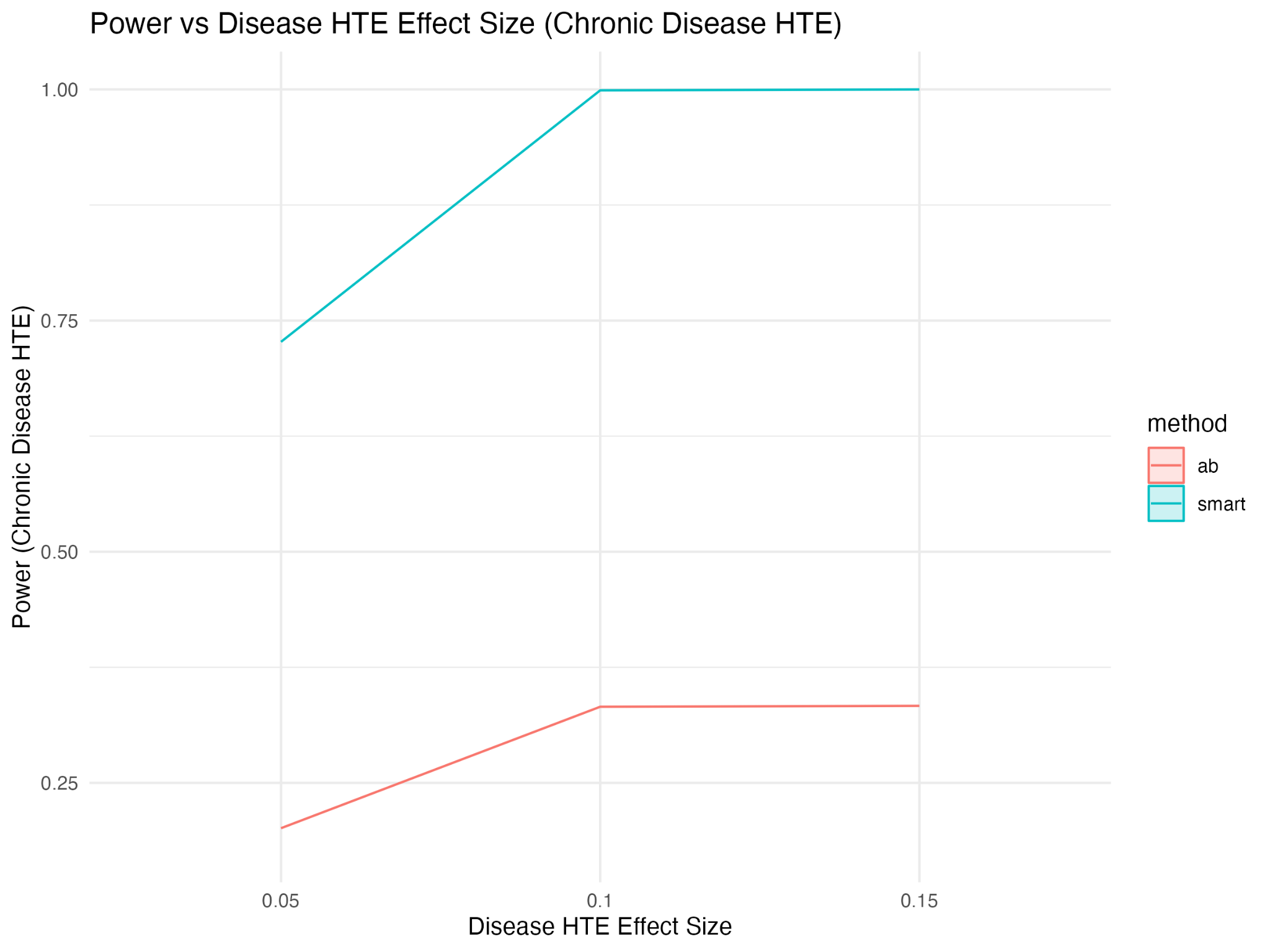


**Supplementary Figure 2:** Comparison of the performance of the A/B test and SMART trial under these conditions to see if the SMART trial's advantage in detecting the disease HTE becomes more pronounced as the population became more homogeneous. We ran simulations with decreasing demographic variations in later stages of the SMART trial. As the SMART trial's advantage in detecting the disease HTE remained the same as the base case (main text Figure 1) as the effect size increased, the results suggest that the population homogeneity did not contribute to the SMART trial's performance. HTE: Heterogeneous treatment effect.


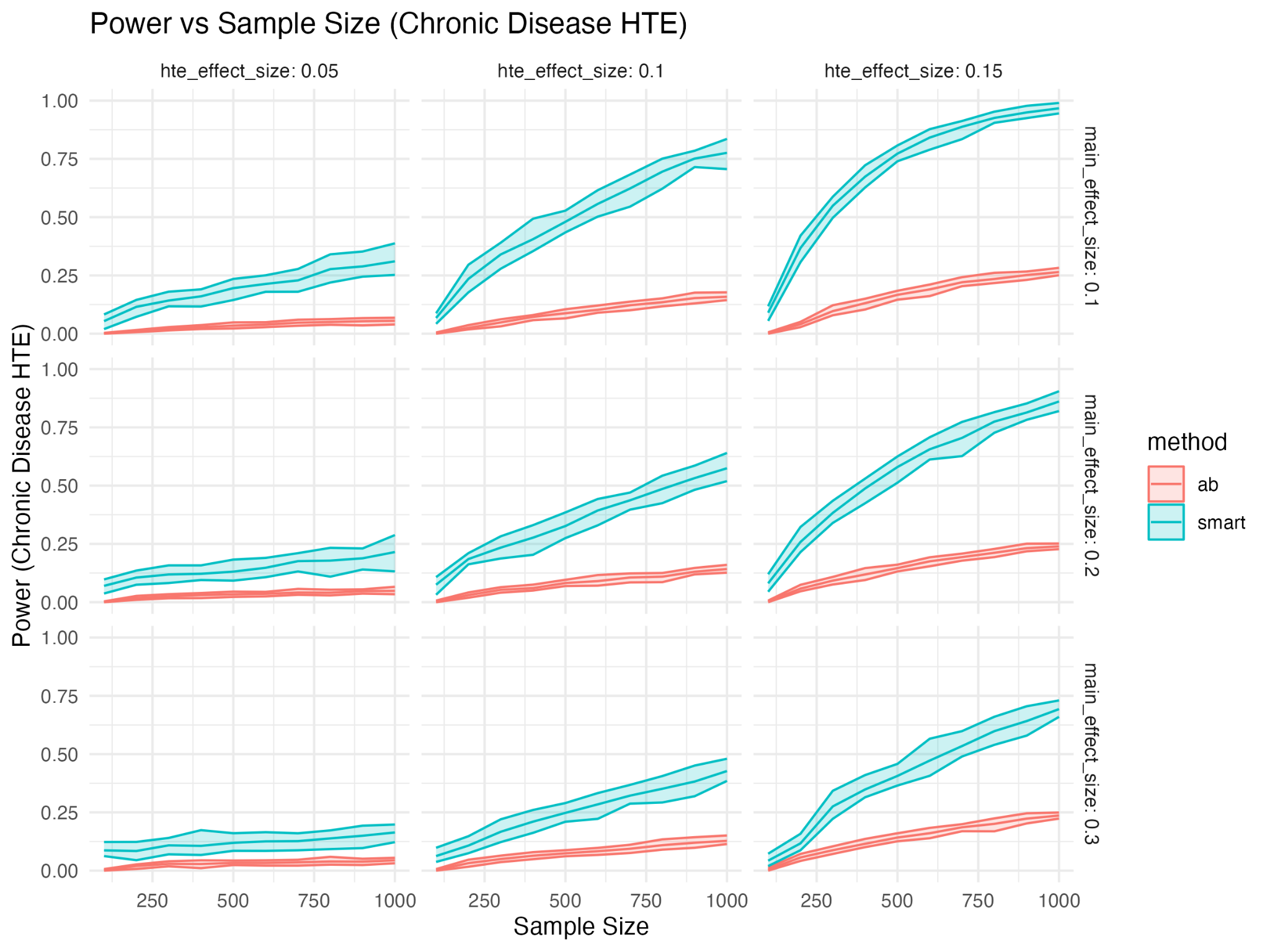

Supplement: Supplementary file 1 — Supplemental Information [file 41746_2024_1330_MOESM1_ESM.docx]
